# Liraglutide preconditioning attenuates myocardial ischemia/ reperfusion injury via homer1 activation

**DOI:** 10.18632/aging.202429

**Published:** 2021-02-01

**Authors:** Xiangrong Cui, Hongping Liang, Chonghua Hao, Xuan Jing

**Affiliations:** 1 Reproductive Medicine Center, Shanxi Maternal and Child Health Care Hospital, Affiliated of Shanxi Medical University, Taiyuan 030001, China; 2 Clinical Laboratory, Shanxi Provincial People’s Hospital, Affiliated of Shanxi Medical University, Taiyuan 030001, China

**Keywords:** homer1, liraglutide, ischemia/reperfusion injury, calcium homeostasis

## Abstract

Myocardial infarction (MI) is one of most common cardiovascular diseases, and ischemia/reperfusion (I/R) injury is one of the risk factors for severe myocardial injury and dysfunction, even leading to high mortality of myocardial infarction. Liraglutide, a novel glucagon-like peptide 1 (GLP-1) analogue, has been reported to reduce cardiac rupture and infarct size and improve cardiac function in normal and diabetic rodents, however, the mechanisms of liraglutide on cardiomyocytes is not clear. The current research was designed to investigate the hypothesis that liraglutide would protect cardiomyocytes through regulating homer1 expression under hypoxia/reoxygenation (H/R) condition. The results of the present study indicated liraglutide reduced hypoxia-reoxygenation induced cell death and attenuated intracellular calcium overload in H9C2 cardiomyocytes under H/R condition. Moreover, liraglutide significantly increased the Homer1 protein expression, and this protection might be related to Homer1-dependent regulation of endoplasmic reticulum (ER) calcium homeostasis. Taken together, liraglutide protects H9C2 cell against H/R induced cell injury, and this protective effect may inhibit intracellular calcium overload to some extent, through Homer1-dependent regulation of ER calcium homeostasis.

## INTRODUCTION

Acute myocardial infarction (AMI) is most commonly associated with acute coronary artery occlusion and remains a major cause of high human morbidity and mortality worldwide [1]. Rapid reperfusion, which is usually achieved by intra-arterial thrombolysis or percutaneous transluminal coronary intervention, is clearly essential to salvage jeopardized myocardium from coronary occlusion [2]. However, reperfusion itself may lead to further cardiac dysfunction, known as myocardial ischemia/reperfusion injury (MI/RI), after which the restoration of blood flow leading to ischemic tissue is not optimal [3]. Therefore, it is essential to investigate the mechanisms of myocardial ischemia/reperfusion injury to elucidate potential prevention strategies.

Homer proteins, known as scaffold proteins which are located at the postsynaptic region, prominently interact with postsynaptic density proteins [4]. Homer proteins can be divided into three subtypes (Homer1, Homer2, Homer3) and several splice variants due to alternative splicing of premature termination of transcription [5]. As a cytosolic adaptor, Homer1 can up-regulate SOCE, playing a central role in Ca^2+^ signaling. Previous studies on Homer mainly focused on the central nervous system (CNS), but for now, more and more researchers began to investigate the role of Homer in cardiovascular disease [6]. Our previous studies demonstrated that Homer1 protects cardiomyocytes against myocardial ischemia induced injury, and we further demonstrated that Homer1 protects cardiomyocytes against I/R injury [7]. Therefore, it is essential to explore a drug that effectively regulates Homer1 expression to protect cardiomyocytes against I/R injury.

Liraglutide is a novel glucagon-like peptide 1analogue, which has been reported to improve cardiac function and reduce infarct size in rodents [8]. Previous studies have shown liraglutide protects cardiomyocytes against I/R injury by inhibiting Ca^2+^ overload and oxidative stress, however, the mechanisms of liraglutide on cardiomyocytes is not clear [9, 10]. The current research was designed to investigate the hypothesis that the GLP-1 analogue liraglutide would protect cardiomyocytes through regulating homer1 expression under hypoxia/reoxygenation (H/R) condition. The results of this study indicated liraglutide reduced death and intracellular calcium overload of H9C2 cell under H/R condition. Moreover, liraglutide significantly increased Homer1 protein level, as well as this protection might be related to ER calcium homeostasis depended on Homer1 regulation.

## RESULTS

### Effects of liraglutide on H/R-induced cytotoxicity

**Figure 1:**
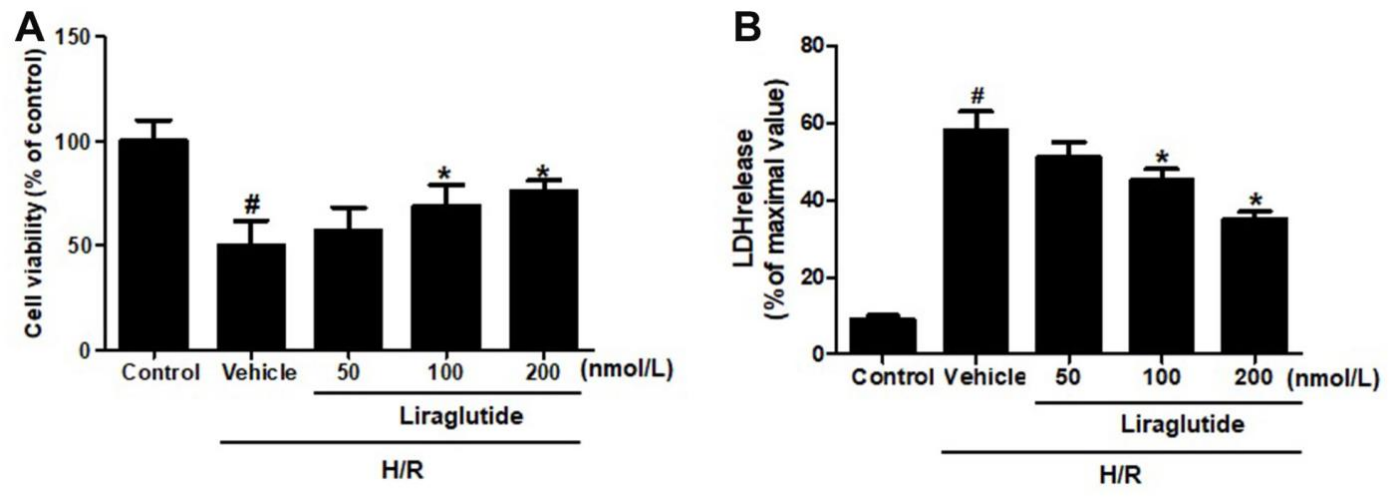
**Effect of liraglutide on H/R-induced cytotoxicity.** H9C2 cells were pretreated with liraglutide in different concentrations (50 nmol/L, 100 nmol/L, 200 nmol/L) 30 min before H/R treatment, and the cell viability (**A**) and LDH release (**B**) were assayed 24 h later.

To explore the protective effect of liraglutide on H9C2 cells, which were pretreated with 50 nmol/L, 100 nmol/L, and 200 nmol/L liraglutide 30 min before H/R treatment. CCK-8 regent was performed to measure the cell viability after H/R treatment. It was revealed that 100 nmol/L and 200 nmol/L liraglutide significantly prevented the decrease of cell viability treated by H/R, although 50 noml/L liraglutide had no effect compared with vehicle group (Figure 1A). We also measured the release of LDH in H/R-treated H9C2 cells, showed a similar protective effect on LDH release that liraglutide inhibited the LDH release at 100 nmol/L and 200 nmol/L, not 50 noml/L (Figure 1B). 200 nmol/L liraglutide was applied in the following experiments.

### Effect of liraglutide on H/R-induced cell death

**Figure 2:**
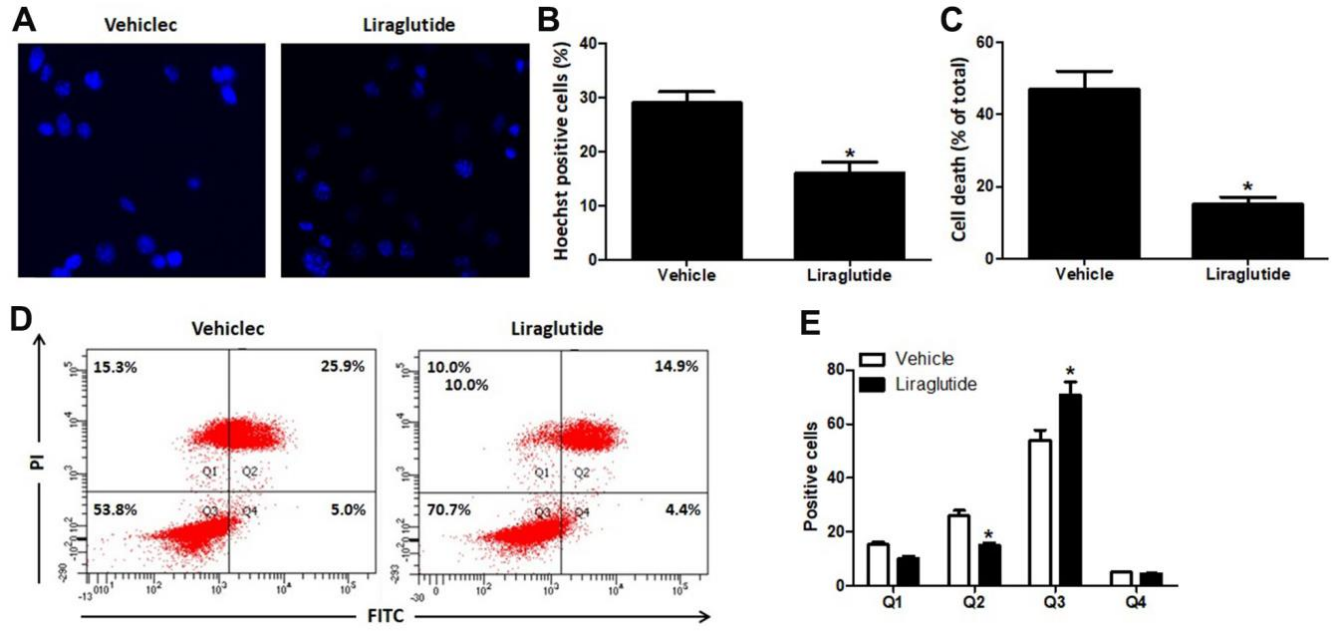
**Effect of liraglutide on H/R-induced cell death.** H9C2 cells were pretreated with 200 nmol/L liraglutide or vehicle 30 min before H/R treatment, and stained with Hoechst 33342 (**A**) 24 h later. The number of Hoechst-positive (**B**) was calculated. Percentage of death cells were counted by Automated Cell Counter (**C**). The number of cells in Q1 (AV-/PI+, The necrotic cells), Q2 (AV+/PI+, the late phase apoptotic cells), Q3 (AV-/PI-, normal cell) and Q4 (AV+/PI-, the early phase apoptotic cells) were also analyzed using Flow cytometry (**D**, **E**).

Hoechst 33342 staining was used to detect H9C2 nuclear damage induced by H/R treatment (Figure 2A, 2B). In vehicle group, H/R treatment induced DNA fragmentation and nuclear chromatin condensation, while 200 nmol/L liraglutide prevented these morphological changes in injured H9C2 cells, suggesting that liraglutide has protective effect on nuclear damage. We further detected the percentage of death cells by Automated Cell Counter (Bio-Rad Laboratories, Hercules Hercules, California, USA), and 200 nmol/L liraglutide significantly decreased the percentage of death cells as compared to vehicle group (Figure 2C). In addition, flow cytometry was also performed to detect the apoptosis of H9C2 cells after H/R treatment (Figure 2D). The results showed 200 nmol/L liraglutide not only increased the number of AV-/PI- cells, but also decreased the number of AV+/PI+ cells, indicating that liraglutide has anti-apoptotic activity (Figure 2E).

### Effect of liraglutide on H/R-induced intracellular calcium overload

**Figure 3:**
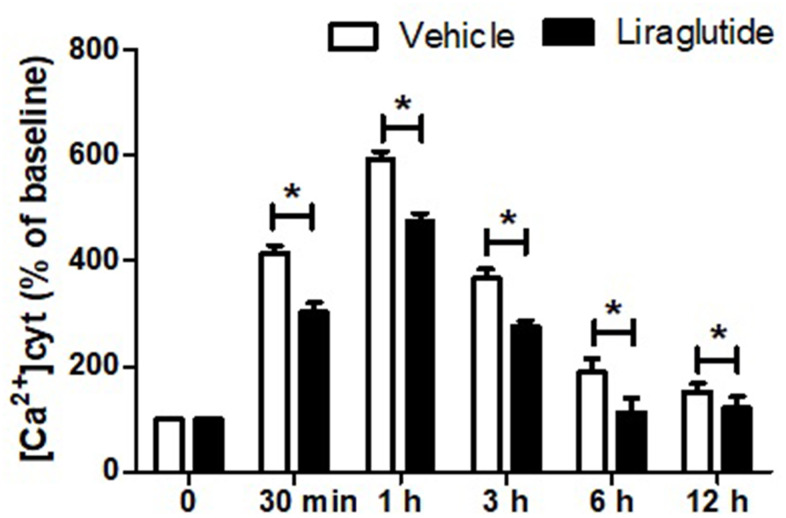
**Effect of liraglutide on H/R-induced intracellular calcium overload.** H9C2 cells were pretreated with 200 nmol/L liraglutide or vehicle 30 min before H/R treatment, and the intracellular Ca^2+^ concentration ([Ca^2+^]_cy_) was measure up to 12 h.

To evaluate the potential role of intracellular Ca^2+^ homeostasis in the protection of liraglutide, we detected [Ca^2+^]_cyt_ using Fura-2-AM. Dynamic changes of [Ca^2+^]_cyt_, expressed as a percentage of the baseline for up to 12 h following H/R injury was shown in Figure 3. H/R injury resulted in a rapid increase of [Ca^2+^]_cyt_ with 1 h, and then slowly returned to baseline after 12 h. Compared with vehicle cells, liraglutide pretreatment significantly reduced [Ca^2+^]_cyt_ at 30 min, 1 h, 3 h, and 6 h, suggesting delayed calcium afflux and inhibited Ca^2+^ overload after H/R treatment.

### Liraglutide promoted expression of homer1 in H9C2 cells against H/R

**Figure 4:**
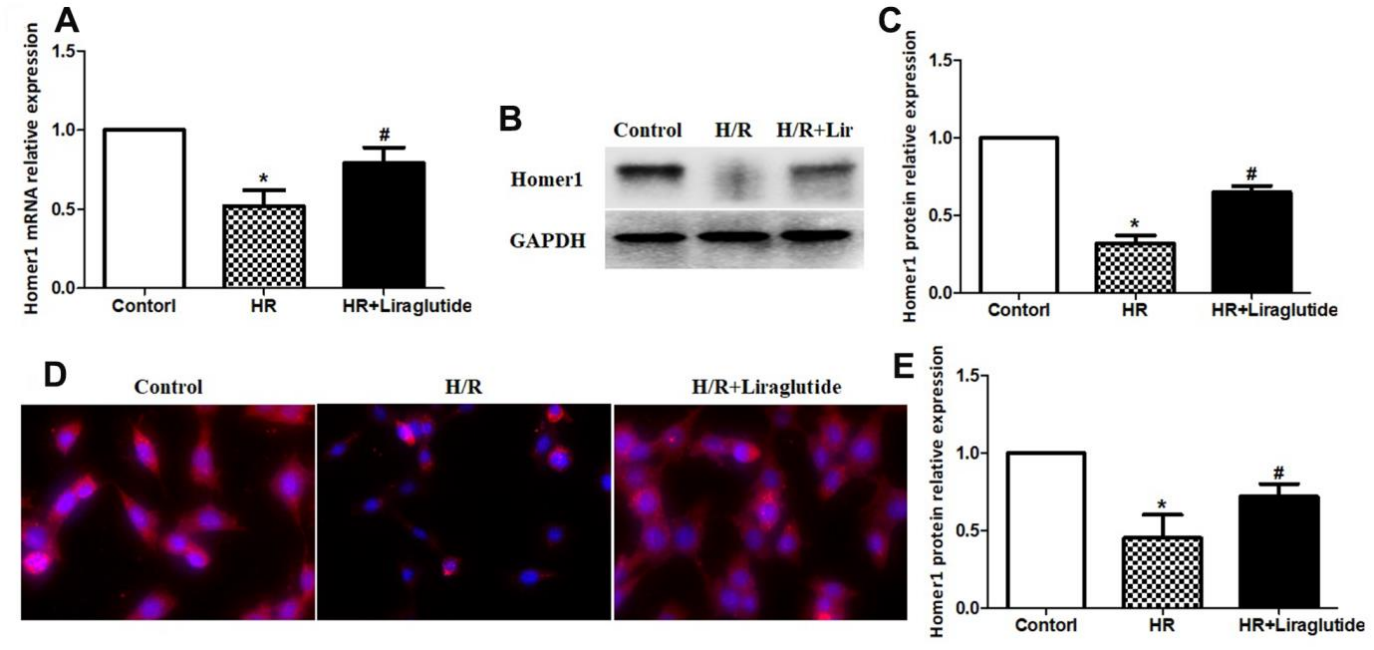
**Liraglutide promoted expression of Homer1 in H9C2 cells against H/R.** H9C2 cells were pretreated with 200 nmol/L liraglutide or vehicle 30 min before H/R treatment, and the expression of Homer1 mRNA (**A**), protein (**B**, **C**), and intracellular localization (**D**, **E**) were examined by RT-qPCR, western blot and immunofluorescence staining.

After 4 h after reoxygenation, we investigated the effect of liraglutide on expression of Homer1 in H9C2 cells by RT-qPCR. The results demonstrated that H/R reduced expression of Homer1, but liraglutide reverse the effect. Pre-treatment liraglutide promoted mRNA expression of Homer1 in H9C2 cells against H/R (Figure 4). To further confirm the H/R inhibition of Homer1, protein expression of Homer1 were examined with western blot and immunofluorescence staining. The results indicated Homer1 protein expression is reduced significantly in H9C2 after 4h of H/R treatment, and liraglutide could reverse the effect too (Figure 4).

**Figure 5:**
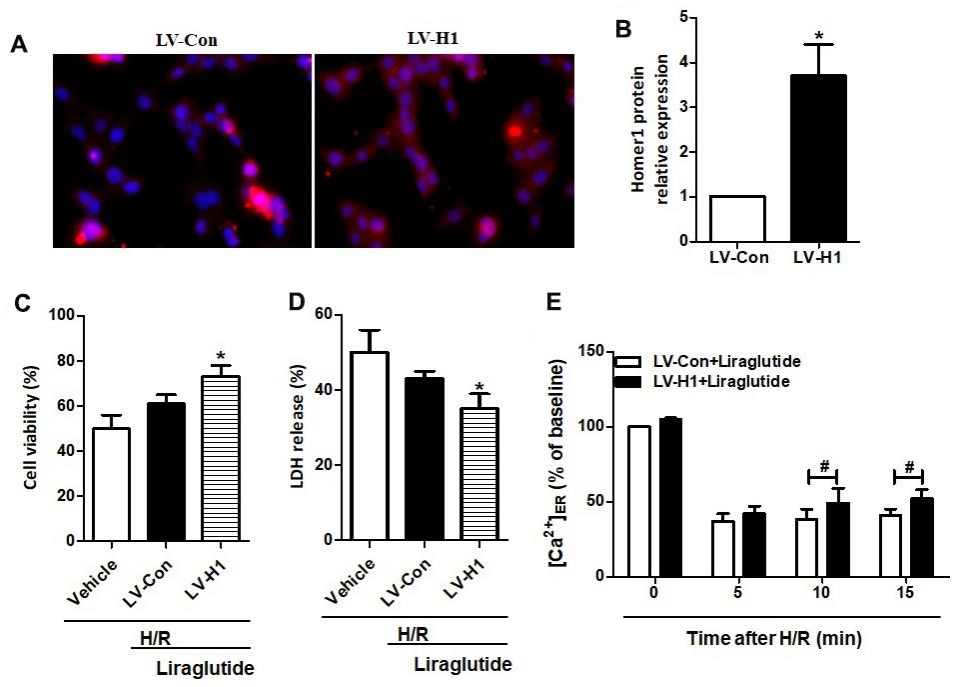
**Effect of Homer1 overexpression on liraglutide-induced cardiomyocyte protection.** H9C2 cell were transfected with LV-H1 or LV-Con for 72 h before H/R treatment, and the expression of Homer1 was examined by immunofluorescence staining (**A**, **B**). The cell viability (**C**) and LDH release (**D**) were assayed 24 h later, and the Ca^2+^ concentration in ER ([Ca^2+^]_ER_) was measured at 5, 10 and 15 min after LV-Con and LV-H1 (**E**).

**Figure 6:**
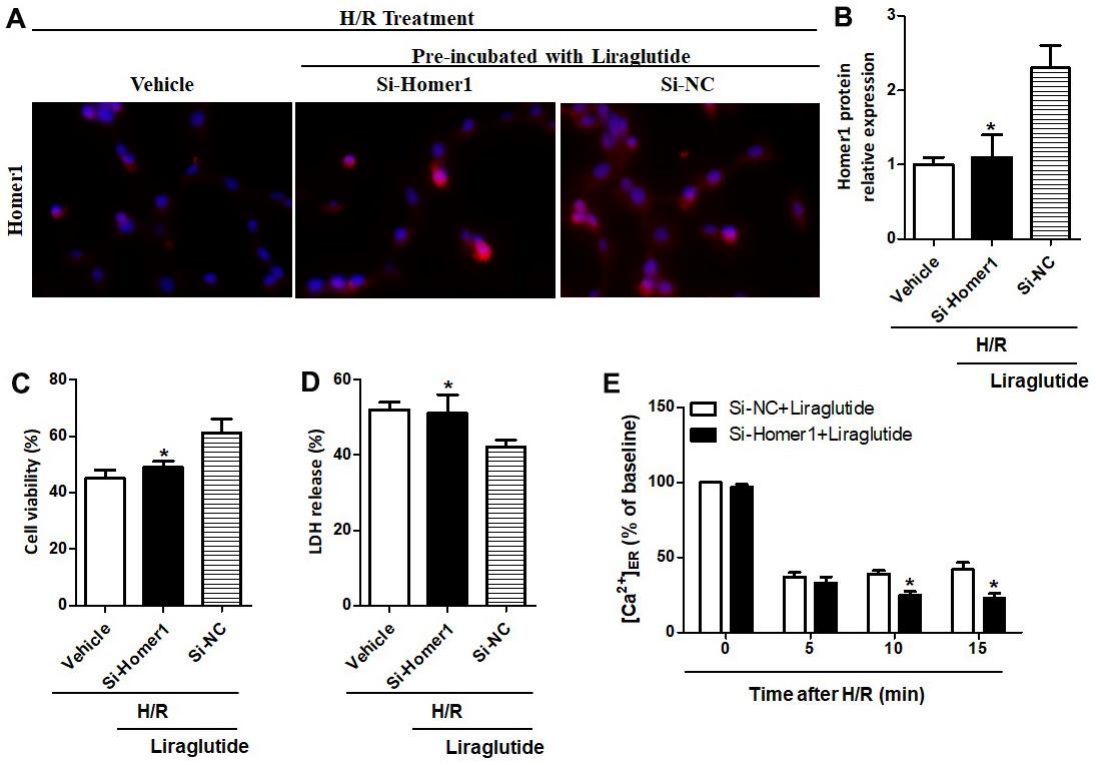
**Effect of Homer1 knock out on liraglutide-induced cardiomyocyte protection.** H9C2 cell were transfected with si-Homer1, si-NC or vehicle for 72 h before H/R treatment, and the expression of Homer1 was examined by immunofluorescence staining (**A**, **B**). The cell viability (**C**) and LDH release (**D**) were assayed 24 h later, and the Ca^2+^ concentration in ER ([Ca^2+^]_ER_) was measured at 5, 10 and 15 min after si-Homer1 and si-NC (**E**).

To further illuminate the relationship between protective effects of liraglutide on H/R-induced myocardial injury and its promoted activity on Homer1 expression, we transiently transfected Homer1 letivirus (LV-H1) and control lentivirus (LV-Con) in H9C2 cells. Immunofluorescence staining suggested that exogenous Homer1 was expressed in H9C2 cells after LV-H1 transfection, and the total level of Homer1 was about 4 times higher than that vehicle cells (Figure 5A, 5B). As revealed in Figure 5C, 5D, liraglutide had a protective effect on H/R-induced cytotoxicity by upregulating cell viability and down-regulating LDH release, which is synergistic with LV-H1 transfection. Furthermore, [Ca^2+^]_ER_ level was significantly increased by LV-H1 transfection as compared on LV-Con transfected H9C2 cells at 10 min and 15 min, suggesting an inhibited ER Ca^2+^ release and promoted calcium recovery in Homer1 upregulated H9C2 cells following H/R treatment (Figure 5E). In addition, we transfected si-Homer1 and si-NC to inhibit the Homer1 protein expression, and then observed whether it could counteract these cardioprotective effects of liraglutide. Immunofluorescence staining indicated that the expression of Homer1 was remarkably repressed after Homer1 was knocked down (Figure 6A, 6B). As revealed in Figure 6C, 6D, the protective role of liraglutide on H/R-induced cytotoxicity, as revealed by upregulated cell viability and downregulated LDH release, counteracted by si-Homer1 transfection. Furthermore, the level of [Ca^2+^]_ER_ was significantly reduced by si-Homer1 transfection as compared on si-NC transfected H9C2 cells at 10 min and 15 min, further confirming an inhibited ER Ca^2+^ release and counteracted these cardioprotective effects of liraglutide in Homer1 knocked out H9C2 cells following H/R administration (Figure 6E).

## DISCUSSION

Since the phenomenon of ischemia/reperfusion injury was first described in 1960s, the research and technology on solving this clinicopathological problem has been made great progress in the past few decades [11]. The clinical efficacy of reperfusion therapy is greatly limited by the occurrence of myocardial I/R injury [12]. In current clinical practice, only pharmacological preconditioning is a promising strategy with limited scope of application and beneficial so far [13]. Previous research suggested liraglutide preconditioning reduced cardiac rupture and infarct size, and improved cardiac output in mice, however, the effect of liraglutide on cardiomyocytes is not clear [14, 15].

To determine the protect effect of liraglutide in the myocardia I/R injury, H9C2 cells were pre-treated with liraglutide in different concentrations before H/R treatment. Our results revealed that certain concentration of liraglutide significantly prevented the decrease of cell viability, percentage of death cells and LDH, which treated by H/R. Subsequently, we found that liraglutide not only increased the number of AV-/PI- cells, but also decreased the number of AV+/PI+ cells, indicating that liraglutide has anti-apoptotic activity. Hoechst 33342 staining showed that liraglutide prevented the changes of DNA fragmentation and nuclear chromatin condensation in injured H9C2 cells.

Previous studies have revealed that Ca^2+^ overload is the primary stimulators to damage mitochondrial function and induce cardiomyocytes death in H/R condition [16, 17]. Liraglutide was originally described as a regulator, promote excessive Ca^2+^ reflux to the sarcoplasmic reticulum and prevent Ca^2+^ release from the SR, finally restoring intracellular calcium homeostasis [10, 18]. Our present study further demonstrated that liraglutide at the concentration of 200 nmol/L significantly decreased the intracellular calcium overload induced by H/R treatment.

Homer proteins are considered to be part of the complex scaffolding of proteins that comprise the PSD, which regulate intracellular calcium homeostasis, such as inositol 1,4,5-triphate receptor (IP3R) and ryanodine receptors [19, 20]. The involvement of Homer1 in some cardiovascular disease conditions has been demonstrated in our and previous studies [7, 21, 22]. In this study, we revealed that pre-treatment liraglutide promoted mRNA and protein expression of Homer1 in H9C2 cells against H/R. To further illuminate the relationship between protective effects of liraglutide and its promoted activity on Homer1 expression, H9C2 cells were transfected with LV-H1 and LV-Con, respectively. We found that the protective of liraglutide on H/R-induced cytotoxicity, cooperated with LV-H1 transfection. Moreover, our results demonstrated that Liraglutide could promote endoplasmic reticulum calcium reflux through Homer1, thus maintaining intracytoplasmic Ca^2+^ homeostasis.

In summary, our current study demonstrated that liraglutide protects H9C2 cells against H/R induced cytotoxicity. Furthermore, these protective effects were partly dependent on Homer1 to attenuate calcium overload and ER calcium release. These findings provide more evidence for liraglutide to be performed to protect cardiac function in patients under H/R injury, however, it need more further research to elucidate the molecular mechanism underlying the protective effect of liraglutide on modulation of calcium homeostasis in cardiomyocytes against H/R injury.

## MATERIALS AND METHODS

### H9C2 cell culture

H9C2 cells (Chongqing Medical University, Chongqing, China) were cultured in DMEM/F12 medium added with 10% FBS and incubated at 37° C in 5% CO_2_ and 95% humidity.

### H/R injury induction *in vitro* and liraglutide treatment

We followed previously described methods to generate a cell model of H/R *in vitro* [23]. When H/R was treated, H9C2 cells were laid out in the glucose-free DMEM/F12 medium and cultured for 24 h in a hypoxic condition (1% O_2_, 94% N_2_ and 5% CO_2_) at 37° C. Following hypoxic injury, cells were placed in reoxygenation in atmosphere condition containing 5% CO_2_-0.5% humidity for 4 h. Untreated normoxic cells were cultured as a negative control. For liraglutide treatment, cultured cells were pre-incubated with liraglutide in different concentrations (50 nmol/L, 100 nmol/L and 200 nmol/L) for 30 min before H/R treatment.

### Cell transfections

The Homer1 lentivirus (LV-H1) overexpressing vectors, along with the control lentivirus (LV-Con) were constructed by GenePharma (Shanghai, China). The siRNAs for Homer1 (si-Ho-mer1), as well as their negative controls (si-NC), were also constructed and purchased from GenePharma (Shanghai, China). miRNAs and siRNAs were conducted by Lipofectamine 2000 reagent (Invitrogen, Carlsbad, USA) for transfection. A total of 1×10^6^ H9C2 cells were infected with 1ml viruses and cultured for 24h. After 48h, the cells were stably overexpressed Homer1, as well as knocked down Homer1, which were screened by puromycin (1μg/ml).

### Cell viability assay

Cell viability of H9C2 cells were detected by Cell Counting Kit (CCK-8). Briefly, H9C2 cells treated with different treatments were collected and cultured with 10 μl CCK-8 reagent at 37° C for 1h. Cell viability was measured at 450 nm with enzyme reader.. All measurements were performed with 8 technical replicates.

### Lactate dehydrogenase release assay

The release of lactate dehydrogenase (LDH) into the medium was detected by Lactate Dehydrogenase Assay Kit (Abcam, Cambridge, MA, USA). 50 μl of supernatant from each cell culture medium was collected for subsequent examination.

### Hoechst 33342 staining

For Hoechst 33342 staining, H9C2 cells were laid onto cover glass slides with Polylysine attached at a density of 3×10^5^ cells/cm^2^. Subsequent, cells were incubated with Hoechst 33342 (10 μg/ml, Thermo Fisher Scientific Waltham, Massachusetts, US) for 10 min and obtained fluorescence through a laser confocal microscope (Nikon Corporation, Japan).

### Cell cycle and flow cytometry

Flow cytometer (BD FACSCanto) was performed to investigate the cell cycle distribution. Each group of cells were collected and placed into Eppendorf (EP) tubes at a density of 1.5×10^5^ cells/tube. Subsequent, 70% ethanol was used to fix cells for 24 h at 4° C. Finally, the cells were measured by Cell Cycle and Apoptosis Analysis Kit (Beyotime Biotechnology, Shanghai, China), incubated with RNase (1 mg/ml; Beyotime Biotechnology, Shanghai, China) for 30 min at room temperature.

### Calcium imaging

Fura-2-AM (Beyotime, Shanghai, China) was performed to measure intracellular Ca^2+^ concentration in ER ([Ca^2+^]_ER_) and cytoplasm ([Ca^2+^]_cyt_) as previously described [24]. H9C2 cells were incubated on glass slides added with 5μM fura-2-AM for 30 min. Subsequently, cells were incubated in imaging chamber containing PBS and added with 20 mM glucose at room temperature. Fura-2 binding with Ca^2+^ can produce strong fluorescence under the excitation light of 330-350 nm, while it will lead to fluorescence attenuation under the excitation light of 380 nm. Thus, the ratio of fluorescence of 340 nm and 380 nm can be used to detect intracellular calcium concentration. Using multifunctional luciferase microplate reader (Sp-max 3500FL; Shanghai Spectrum Instruments Co.,LTD), fura-2 were excited at 340 and 380 nm and the emission fluorescence was recorded at 510 nm.

### Immunofluorescence

Coverslips containing attached H9C2 cells were fixed with 4% formaldehyde for 15 min, permeabilized with 0.25% Triton X-100 for 20 min, and closed antibodies with PBS-BSA for 1 hour. Slides were incubated with Homer1 rabbit polyclonal primary antibody (1:200, Proteintech Group, Inc, Wuhan, Hubei, China) at 4° C in overnight. In addition, cells were cultured with goat resist rabbit secondary antibodies (1:200; Affinity Biosciences, Cambridge, Uk) for 1 h. Then, DAPI stained the nuclei and detected fluorescence.

### RT-qPCR analysis

mRNA was reverse-transcribed into cDNA using BeyoRT™II First Strand cDNA Synthesis Kit (Beyotime Biotechnology, Shanghai, China). Real-time PCR was performed on a Real-time PCR system (CFX96, Bio-Rad Laboratories, Hercules, California, USA) using FastFire qPCR PreMix (SYBR Green) (Tiangen Biotech, Beijing, China). All the assays were independently repeated for 3 times. Homer1 mRNA was amplified through polymerase chain reaction (forward, 5'-ATAGCACCATCACTCCAAA-3'; reverse, 5'-GAATCCCAGTCCATAAACA-3'). GAPDH (forward, 5'-ACCACAGTCCATGCCATCAC-3'; and reverse, 5'-TCCACCACCCTGTTGCTGTA -3') was used as the internal reference gene for normalization of mRNA levels [25].

### Western blot analysis

Proteins (40 μg) from H9C2 cells were separated by SDS-PAGE gel electrophoresis, which was followed by electroblotting onto PVDF membranes (EMDMillipore). The blot was probed by using respective primary antibodies including Homer1 (1:1000), GAPDH (1:2000; Proteintech Group, Inc. Wuhan, China). The bands were analyzed by Hypersensitive ECL chemiluminescence kit (BeyoECL Plus, Beyotime, Shanghai, China).

### Statistical analysis

GraphPad7.0 software (GraphPad Software, Inc.) was performed to analyze data with independent sample Student’s t-test or one-way analysis of variance analysis of variance test (ANOVA) with post hoc contrasts by Student-Newman-Keuls test (*P*<0.05). All values are reported as mean ± standard deviation (SD) of the mean.
